# Ensemble‐Based Machine Learning Models for Classifying Intervention‐Related Psychological Outcome Patterns in Menopausal Women Treated With *Pimpinella anisum*

**DOI:** 10.1002/brb3.71787

**Published:** 2026-10-09

**Authors:** Md Belal Bin Heyat, Arshiya Sultana, Attiq Ur Rehman, Arfa Begum, Mohamad Sawan

**Affiliations:** ^1^ CenBRAIN Neurotech Center of Excellence, School of Engineering Westlake University Hangzhou China; ^2^ Integrated‐on‐Chips Brain‐Computer Interfaces Zhejiang Engineering Research Center Westlake University, Hangzhou Zhejiang China; ^3^ Department of Amraze Niswan Wa Ilmul Qabalat, National Institute of Unani Medicine Ministry of AYUSH Bengaluru India; ^4^ Department of Cyber Security, School of Cyber Science and Engineering Huazhong University of Science and Technology Wuhan China; ^5^ Electrical Engineering, Polytechnique Montreal Canada

**Keywords:** AI for female disorder, AI for medicine, artificial intelligence, female disorder, machine learning, menopause, psychological disorder

## Abstract

**Background:**

Menopause, a natural biological transition in the life of women, is often associated with significant psychological disturbances such as depression, anxiety, and stress. However, early identification and management of psychological disorders in menopausal women remain under‐addressed due to limited specialist access and a lack of systematic screening approaches.

**Purpose:**

This study aimed to develop ensemble‐based machine learning (ML) models to classify intervention‐related clinical and psychological outcome patterns in menopausal women using clinical and questionnaire‐based data collected during a controlled intervention study.

**Method:**

Psychological and menopausal symptoms were assessed using standardized tools, including the Depression Anxiety Stress Scale‐21 (DASS‐21), modified Kupperman index (MKI), vaginal health index (VHI), and menopause symptoms treatment satisfaction questionnaire (MS‐TSQ). Statistical analyzes were performed using chi‐square tests and independent *t*‐tests to evaluate group differences. Three ensemble classifiers, random forest (RF), gradient boosting (GB), and AdaBoost (AB), were evaluated using repeated cross‐validation.

**Result:**

Among these, the RF classifier achieved the best performance with a sensitivity of 93.3%, a specificity of 90.0%, an accuracy of 91.7%, a precision of 90.3%, and an F1‐score of 91.8%.

**Conclusion:**

These results indicate that ensemble ML models, particularly RF, can serve as effective supplementary tools for psychological disorder risk stratification in menopausal care, aiding clinicians in decision‐making. However, these models are intended to complement, not replace, clinical assessments.

## Graphical abstract

This study develops an ensemble‐based machine learning approach to detect and predict psychological disorders in menopausal women using clinical and questionnaire data. Random Forest achieved the best performance. The framework supports early risk identification and integrates statistical validation with Unani‐based and home remedy interventions to assist clinical decision‐making.

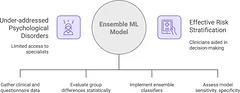

## 1 Introduction

Menopause is a significant life event, signalling the end of a woman's reproductive years. It typically occurs between the ages of 45 and 55, with estrogen and progesterone levels decreasing as part of the natural aging process. The final menstrual period happens typically around age 51 (Kuck and Hogervorst 2024). Menopausal symptoms differ extensively among women, varying from sleep disturbances, anxiety, depression, stress, hot flashes, brain fog, night sweats, urogenital symptoms, and impact on work productivity and daily life activities (Nappi et al. 2023). Women experiencing severe menopausal symptoms often exhibit a sharp level of presenteeism compared to those who do not experience such symptoms (Whiteley et al. 2013).

Research indicates that the likelihood of anxiety and depression in menopausal women is approximately 12.62% and 25.99%, respectively. This data highlights the variability and complexity of emotions experienced during this stage of life (Liu et al. 2023). Psychological symptoms, such as anxiety and depression, are common during the menopausal period (Kuck and Hogervorst 2024). The connection between menopausal symptoms and respective stages of menopause is still unclear. Some studies suggest that these symptoms may be influenced more by psychosocial factors, such as career and family issues, rather than hormonal changes (Mulhall et al. 2018). Additionally, current research examining the link between menopausal status and symptoms like depression has yielded contradictory results (Mulhall et al. 2018). Furthermore, the current study reveals that higher rates of psychological complaints, such as depression, are observed in premenopausal women compared to those who are postmenopausal (Bromberger et al. 2007). A longitudinal study found that transitioning to menopause was associated with increased levels of anxiety in women who had no history of anxiety during their premenopausal phase. Additionally, there is evidence suggesting that panic attacks may become more prevalent during postmenopause.

The clinical manifestations of the menopausal syndrome include somatic symptoms, urogenital problems, vasomotor symptoms, sleep disturbances, sexual dysfunction, and mood disorders. In addition, some other symptoms and conditions, such as palpitations, forgetfulness, restless legs, muscle and joint pains, osteoporosis, and dyslipidaemia, are associated with it. Menopausal women are also at a comparatively higher risk for hypertension, memory loss, and diabetes. Thus, menopausal syndrome significantly impacts women's quality of life. Herbal medicine is estimated to be used by approximately four billion people, which accounts for about 80% of the world's population (Ekor 2014). Additionally, around 22% of women seeking treatment for depression choose to consult a naturopath or herbalist (Casteleijn et al. 2019). Numerous herbal remedies and traditional Unani medicines have been studied to assess their respective effectiveness in treating anxiety and depression (Georg Jensen et al. 2024). For instance, chamomile has demonstrated a significant reduction in anxiety symptoms and is well‐tolerated, even at higher doses compared to a placebo (Saadatmand et al. 2024). Echinacea has been shown to cause a decrease in anxiety over a short period (3 days) (Haller et al. 2025). Passionflower has also shown promising results in reducing anxiety symptoms across multiple clinical trials (La Tempa et al. 2025). However, it is worth noting that many studies on herbal medicines for anxiety have been short‐term, open‐label, or inadequately reported (Casteleijn et al. 2019).

The Unani medicine, rooted in the theory of humors, addresses various illnesses classified by symptoms (Itrat 2020). Renowned Unani scholars, such as Avicenna and Rhazes, discussed conditions like amenorrhoea and menopause. Menopause is characterized by a shift in temperament toward coldness, leading to the natural cessation of menstruation. This phase of shift in temperament is often accompanied by headache, hirsutism, fatigue, insomnia, anxiety, depression, myalgia, joint pain, and nervousness. Classical Unani literature documents various medicinal plants and compound formulations for alleviating these menopausal symptoms. Classical texts like *Rhazes's Liber Continens, Avicenna's Canon* of Medicine (Saadatmand et al. 2024), *Azam Khan's Muhit‐e‐Azam*, and *Najmul Ghani's Khazain‐ul‐Advia* describe the use of *Pimpinella anisum* for disorders attributed to excess black bile (*Sauda'*). These include melancholia, headache, flatulence, palpitation, and certain neurological conditions (Mosavat et al. 2019). These studies suggest their potential role in balancing humoral imbalances that are often exacerbated during the menopausal transition. *Pimpinella anisum* (anise) holds particular significance due to its well‐recognized properties in managing psychological and somatic symptoms associated with menopause.

*Pimpinella anisum* L. [*Apiaceae*], or *Anisoon* (Unani), is still recognized in Unani medicine for its therapeutic benefits in neurological, gastrointestinal, and menopausal symptoms (Farahmand et al. 2021). This herb typically grows to 30–50 cm, bearing clusters of small white flowers that develop into greenish to yellow seeds (Sun et al. 2019). Native to the Mediterranean basin, *P. anisum* is widely cultivated in Iran, India, and Turkey for its aromatic fruits and seeds (Ali et al. 2017; Shahamat et al. 2014). It has emmenagogue, anti‐inflammatory, carminative, diuretic, analgesic, appetiser, sedative, and galactagogue and hepatoprotective (Jamshidzadeh et al. 2015) as well as aphrodisiac properties (Khan 2018; Nahidi et al. 2012), which have also been confirmed in animal experiments (Asadollahpoor et al. 2017; Aiswarya et al. 2018). Anise has been found to have beneficial effects on diseases like dysmenorrhea, hot flashes, diabetes, hyperlipidemia, and gastrointestinal diseases (Mosavat et al. 2019). Scientific studies have proven *P. anisum* has analgesic, anti‐inflammatory, antidepressant (Shahamat et al. 2014; Wang et al. 2018), anxiolytic (Es‐safi et al. 2021), estrogenic, sedative, antioxidant, anticonvulsant, and neuroprotective (Karimzadeh et al. 2012) properties.

Although *P. anisum* is not a classical psychedelic, it represents an alternative therapeutic approach targeting menopausal psychological disorders. Its documented neuropsychological effects, including anxiolytic, antidepressant, sedative, and estrogenic properties, contribute to mental health modulation. Thus, while pharmacologically distinct from classical psychedelics, *P. anisum* aligns with the Special Issue's broader focus on innovative interventions for mental health.

The application of artificial intelligence (AI) is growing in healthcare sectors (Munir et al. 2025). Machine learning (ML) and deep learning techniques are tools for AI applications (Bin Heyat et al. 2024). Pande and Chaki (2025) used a hybrid model based on ML and deep learning for brain tumor classification. Latiff et al. (2025) applied the ML model to detect voice disorders. Swapno et al. (2025) detected skin cancer using ML techniques. Makala and Kumar (2025) used clinical data to classify and predict glaucoma using deep learning and ML techniques. Many of the research and review articles suggested that ML and deep learning techniques are used in the detection, prediction, and classification of psychological disorders such as mental stress (Bin Heyat et al. 2022), mental fatigue (Parveen et al. 2025), anxiety (Akhtar et al. 2024), and depression (Bin Heyat et al. 2025). Li et al. (2024) demonstrated the effectiveness of professional suggestion in depression detection using multimodal network techniques. He, Guo, Tiwari, Pandey, et al. (2022) estimated a depression scale from facial expressions using a three‐dimensional convolutional neural network. Additionally, they also used deep learning to analyze depression using video data He, Guo, Tiwari, Su, et al. (2022). Im et al. (2024) used heart rate variability during intervention for the treatment of post‐traumatic stress disorder. Wu et al. (2022) used a wearable sensor for stress monitoring. Surantha et al. (2021) developed an ML model for sleep stage classification using physiological signals. Additionally, W. Chen et al. (2025) used a small dataset to detect the high‐frequency oscillations using a physiological signal. Biswas et al. (2025) used AI models to predict mental disorders.

ML models were used to analyze traditional therapies such as Unani medicine (Sultana et al. 2023) and yoga (Pal et al. 2022), using clinical and experimental datasets. Sultana et al. (2023) examined the effectiveness of Unani medicine in treating female disorders. While others reviewed randomized controlled trials on herbal remedies for premenstrual syndrome (PMS), focusing on oxidative stress and inflammation (Sumbul et al. 2024; Sultana et al. 2022). Qayyum et al. (2023) highlighted the benefits of psyllium and linseeds for the treatment of pelvic inflammatory disease. Fazmiya et al. (2024) used ML to evaluate the acacia gum and camphor suppositories for managing heavy menstrual bleeding, showing therapeutic potential. These findings emphasize AI's role in advancing Unani medicine and improving women's health. From a theoretical perspective, the adoption of AI tools in healthcare can also be interpreted through established technology adoption frameworks such as the Technology Acceptance Model (TAM), the Technology–Organization–Environment (TOE) framework, and institutional theory. These frameworks emphasize factors such as perceived usefulness, perceived ease of use, organizational readiness, and environmental influences that affect the adoption of new technologies. In the context of this study, the proposed ML‐based framework can be viewed as a clinical decision‐support tool that may assist healthcare providers in identifying psychological symptoms among menopausal women. By improving diagnostic support and decision‐making efficiency, such AI‐driven systems may contribute to the broader adoption of digital health technologies in clinical practice.

This study aimed to evaluate the effects of the herbal intervention and to analyze intervention‐related clinical and psychological outcome patterns using ensemble‐based ML models. Clinical and questionnaire‐derived measurements were analyzed using random forest (RF), gradient boosting (GB), and AdaBoost (AB) classifiers to distinguish patterns associated with the intervention and control groups (CGs). The ML analysis provides a data‐driven assessment of treatment‐related outcome patterns and the potential effectiveness of *P. anisum* in menopausal care. Because the analysis incorporates follow‐up measurements collected after treatment, the models are interpreted as classifiers of intervention‐related outcomes rather than prospective predictors of future psychological disorders. The main contributions of this study are:

- to assess psychological and menopausal symptoms in menopausal women using standardized clinical and questionnaire‐based instruments,
- to evaluate the effects of a “*Pimpinella anisum*”‐based intervention using statistical analyzes,
- to develop and compare ensemble‐based ML classifiers for distinguishing intervention‐related clinical and psychological outcome patterns,
- to evaluate the performance of RF, GB, and AB models for analyzing a relatively small clinical dataset, and
- to provide preliminary evidence regarding the utility of ensemble learning for analyzing treatment‐related response patterns in menopausal women.

## 2 Materials and Methods

This study involves several key steps, including data collection, implementation of the intervention protocol, application of a statistical model to identify significant features, and application of an ensemble‐based ML algorithm to the questionnaire and clinical data for prediction, as illustrated in Figure 1.

**FIGURE 1:**
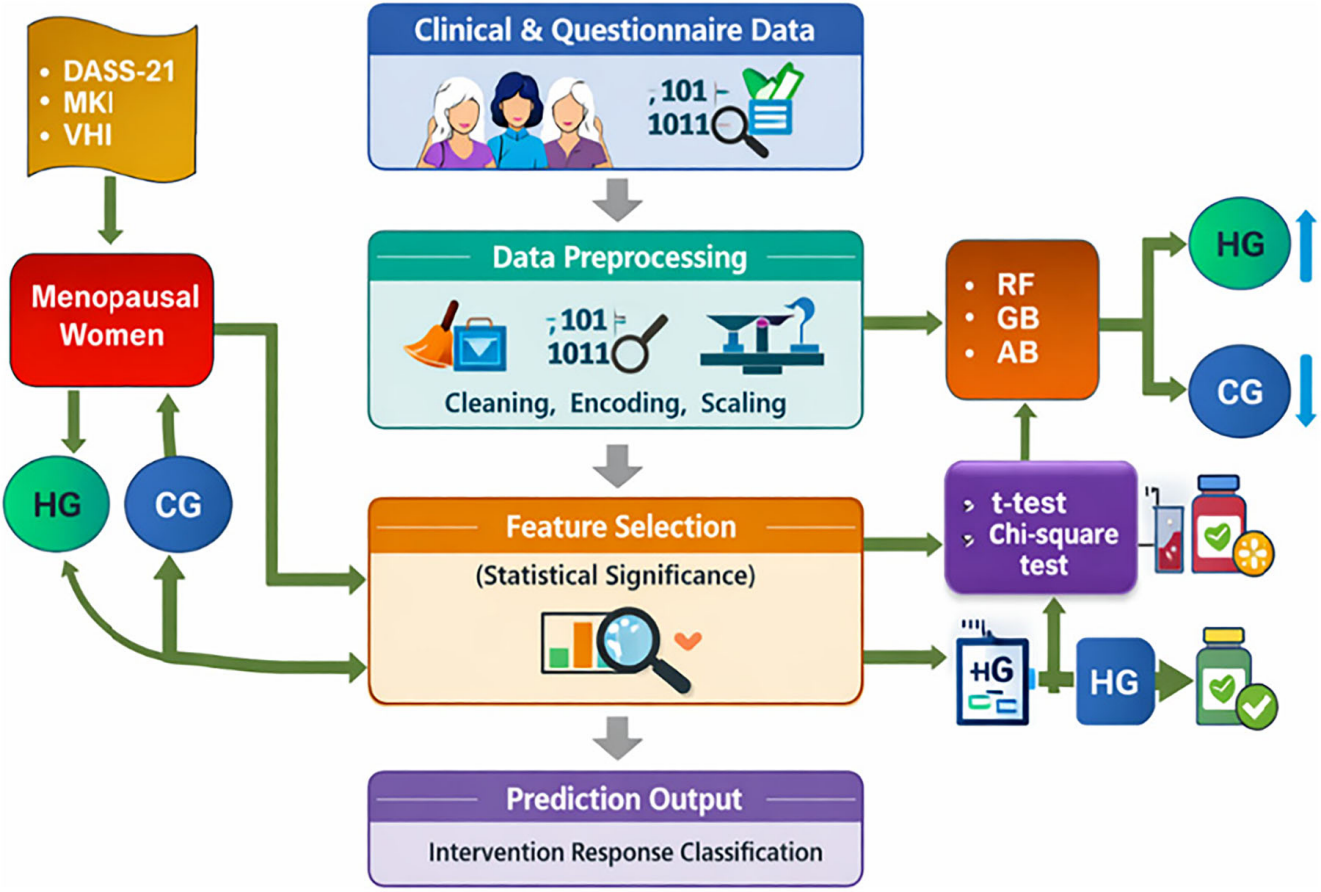
Block diagram of the proposed study.

Figure 1 illustrates the conceptual framework and analytical workflow of the proposed study. The framework outlines the process beginning with data collection from clinical and questionnaire‐based assessments, followed by data preprocessing and feature preparation. The processed dataset is then used to train multiple ML classifiers, including RF, GB, and AB models, which are used to the processed dataset was used to train multiple machine‐learning classifiers, including RF, GB, and AB, to classify intervention‐related clinical and psychological outcome patterns observed among the study participants. The analytical workflow therefore focuses on characterizing and distinguishing treatment‐related response patterns rather than prospectively predicting future psychological risk in menopausal women. This framework provides a structured overview of the analytical pipeline used in the study.

### 2.1 Experimental Data Collection

A total of 210 menopausal women were initially assessed for psychological and menopausal symptoms using the Depression Anxiety Stress Scale‐21 (DASS‐21) and modified Kupperman index (MKI). The detailed search strategy and database‐specific search strings are provided in Supporting Information1.

A total of 60 participants met the inclusion criteria and were randomly assigned to either the HG (*n* = 30) or the CG (*n* = 30) group (Begum et al. 2025). This study included women aged 40–60 years who had experienced amenorrhea. Eligible participants were either postmenopausal (amenorrhea for at least 12 months) or perimenopausal (2–11 months of amenorrhea in the past year). All participants exhibited psychological symptoms associated with menopause, assessed using the DASS‐21 scale, and other menopausal symptoms evaluated by the MKI. Women who had major psychological disorders, were undergoing treatment for systemic or endocrine diseases such as uncontrolled hypertension or diabetes mellitus, or had a history of hypersensitivity to drugs were excluded. Additional exclusion criteria included prior hormone replacement therapy, rapidly growing leiomyomas, malignancies, undiagnosed vaginal bleeding, endometrial hyperplasia, surgical menopause, and lactation.

The sample size was estimated based on the mean and SD from the previous study, with *α* = 0.05 and *β* = 0.10. where mean ± SD of total MKI score was *μ*_1_ = 23.47 ± 7.17 and *μ*_2_ = 19.23 ± 6.16 (R. Chen et al. 2018). The following formula was used to calculate the sample size. When calculated for the required improvement in MKI score, the total sample size calculated was 51.77. A total of 60 participants, with an attrition rate of 15%, and the final sample size was considered to be 30 women in each group.

$$\begin{array}{ccc} n & = & {\left(Z_{\alpha /2}+Z_{\beta }\right)}^{2}\times 2\times \sigma^{2}/d^{2}\\ & = & {\left(1.96+1.28\right)}^{2}\times 2\times 6.66^{2}/{\left(23.47-19.23\right)}^{2}\\ & = & 51.77 \end{array}$$

where *n* is the sample size, *d* is the *μ*_1_ − *μ*_2_, *d* is the clinically significant difference, *μ*_1_ is the mean score of MKI of placebo group = 23.47, *μ*_2_ is the mean score of MKI of test group = 19.23, *σ* is the mean standard deviation = 6.66, $Z_{\alpha /2}$ is the Level of significance for 5% this is 1.96, and $Z_{\beta }$ is the power of the study for 90% this is 1.28.

Participants were randomly allocated to either the HG or CG group at a 1:1 ratio using a block randomization method. The randomization involved 15 blocks of size 4, which were generated by online software. To ensure concealment of treatment assignment, the medications were dispensed in non‐transparent aluminum sachets, sequentially numbered and coded. After the screening visit, a face‐to‐face recruitment interview was conducted to gather sociodemographic and clinical information and to perform a general physical examination. Additionally, scores from DASS‐21, MKI, vaginal health index (VHI), and menopause symptoms treatment satisfaction questionnaire (MS‐TSQ) were recorded. Blood samples were collected to assess hematological and biochemical parameters, to exclude general diseases, and to evaluate hepatic and renal safety. Participants were scheduled for visits every month throughout the 2‐month treatment period, with two additional follow‐up visits at 1‐month intervals, during which no intervention was provided. At each visit, participants were asked about any improvements or worsening of their symptoms and were encouraged to report any adverse effects.

It should be noted that this study involves a relatively small cohort (*N* = 60), representing a pilot‐scale investigation. Therefore, while the reported model performance metrics (e.g., accuracy of 91.7% for the RF classifier) indicate promising results, they should be interpreted cautiously. The findings primarily provide preliminary evidence and should be validated in larger independent cohorts before generalization.

### 2.2 Dataset Composition and Feature Engineering

The dataset used for the machine‐learning analysis consisted of records from 60 menopausal women and included clinical assessments and questionnaire‐based measurements. The predictor variables comprised symptom assessment scores and demographic and clinical characteristics. Symptom‐related predictors included measures derived from the DASS‐21, the MKI, the VHI, and the MS‐TSQ, together with relevant clinical and biochemical variables collected during the study. Demographic and clinical predictors included variables such as age, body mass index (BMI), and menopausal status.

Importantly, intervention group assignment was not included in the predictor set. The intervention group variable, distinguishing participants receiving *P. anisum* treatment from those in the CG, was used exclusively as the binary outcome label for the classification task. Before model training, the target label was separated from the feature matrix to prevent direct target leakage.

Before model development, the dataset was pre‐processed to ensure data quality. Missing values, where applicable, were handled according to the study's predefined analytical procedures. Continuous predictor variables were standardized using z‐score normalization, while categorical predictor variables, such as menopausal status, were encoded appropriately. All preprocessing operations were performed within the training folds of the cross‐validation (CV) procedure, and the learned transformations were subsequently applied to the corresponding validation data to minimize information leakage. Given the relatively modest dimensionality of the predictor set, no dimensionality reduction techniques were applied.

#### 2.2.1 Ethics Approval and Consent to Participate

This study was conducted in accordance with the ethical principles outlined in the Declaration of Helsinki for research involving human participants. The study protocol was reviewed and approved by the Institutional Ethics Committee of the National Institute of Unani Medicine (NIUM), Bengaluru, India. The ethics approval reference number is NIUM/IEC/2020‐21/016/ANQ/07. Prior to participation, written informed consent was obtained from all participants after explaining the purpose, procedures, potential risks, and benefits of the study. Participation was entirely voluntary, and participants were free to withdraw from the study at any time without any consequences.

#### 2.2.2 Clinical Trial Registration

The clinical study associated with this research was registered with the Clinical Trials Registry of India (CTRI), which is the official national registry maintained by the Indian Council of Medical Research (ICMR). The trial was conducted at the NIUM, Bengaluru, India, and registered under the CTRI with the registration number CTRI/REF/2022/02/039961.

#### 2.2.3 Statistical and Regression Analysis

To ensure transparency in the statistical modeling process, all variables used in the analysis were clearly operationalized prior to model development. Demographic variables such as age, BMI, and menopausal status were included as clinical predictors, while psychometric variables were derived from validated questionnaires, including the DASS‐21, MKI, and VHI.

Prior to model development, diagnostic tests were performed to evaluate potential multicollinearity among predictor variables. The variance inflation factor (VIF) was calculated for continuous predictors, and variables with high collinearity were carefully examined to ensure model stability.

In addition to the statistical analysis, ML models were evaluated using CV procedures to improve robustness and reduce overfitting. This approach ensured that the predictive performance reported in this study reflects the model's generalization capability rather than overfitting to the training data.

### 2.3 Intervention Protocols

#### 2.3.1 *Pimpinella anisum* Linn Medicine for Herbal Group (HG)

*Pimpinella anisum* fruits were purchased from the local market, and a Botanist from Central Ayurveda Research Institute, India, authenticated the plant material. Under Good Manufacturing Practices (GMP), the medicine was prepared for this study using *P. anisum* fruit at the NIUM, India.

The fruits were dried at room temperature and then cleaned and processed using a pulverising hammer mill. The resulting powder was passed through a mesh screen of size 80 to obtain a fine powder. Note that 667 mg of the sieved powder was encapsulated. Participants were instructed to take three capsules twice daily for 8 weeks. Figure 2a demonstrates the procedure of *P. anisum* medicine design for HG.

**FIGURE 2:**
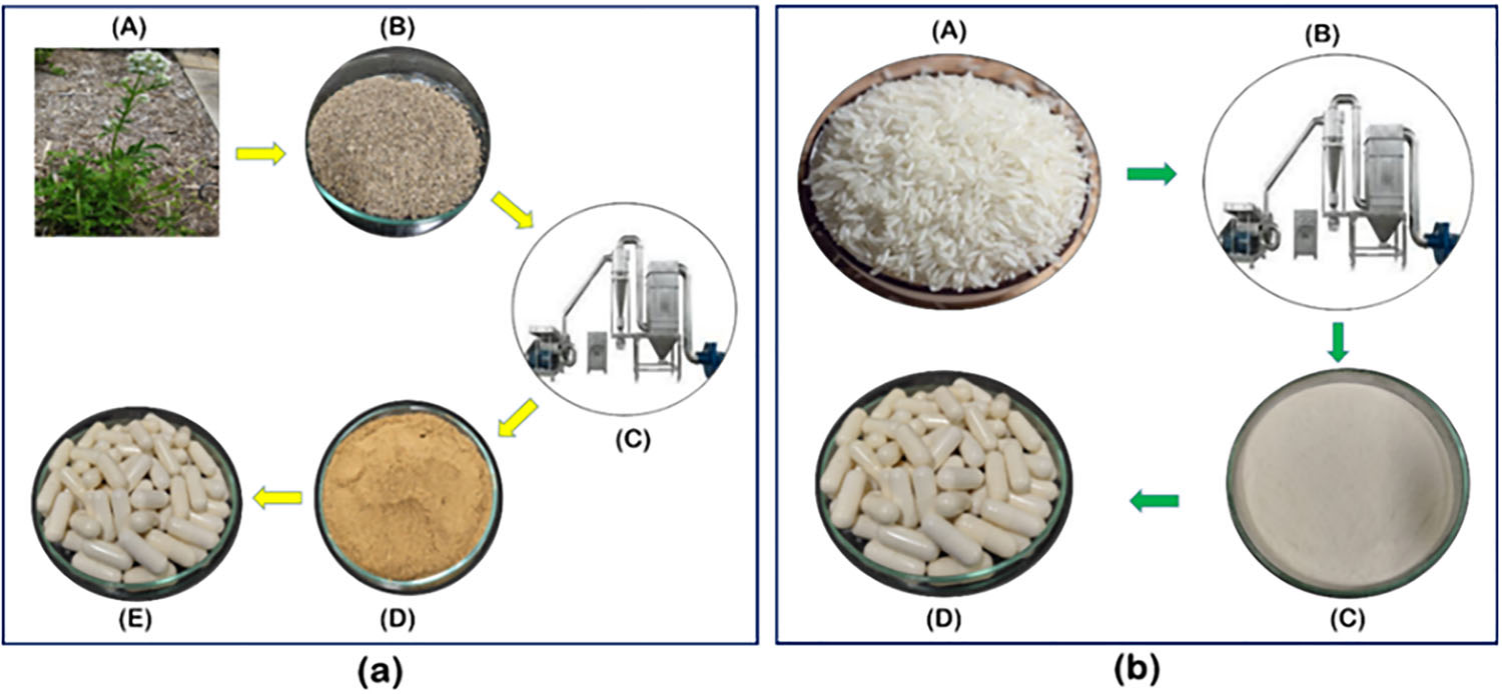
The medication preparation process is based on (a) *Pimpinella anisum* for HG and (b) roasted rice flour for CG.

#### 2.3.2 Roasted Rice Flour for CG

The CG medicine was prepared using rice flour at NIUM, India. The rice was dried at room temperature, cleaned, and processed using a pulverising hammer mill. The resulting fine powder, passed through an 80‐mesh screen, was encapsulated, with each capsule containing 667 mg. Participants were advised to take three capsules twice daily for 8 weeks. The study packs were dispensed sequentially based on the order in which participants entered the study as per randomization codes. The code numbers were not revealed until the trial was completed and the data analysis was conducted. Figure 2b illustrates the drug design procedure for CG, based on roasted rice flour.

Roasted rice flour was selected because it is organoleptically neutral (in terms of taste and smell), pharmacologically inert, and visually identical to the intervention capsules, which helps maintain blinding integrity. Roasted rice flour, a commonly accepted inert substance, has been widely documented as a safe placebo material in herbal and nutraceutical intervention studies.

### 2.4 Questionnaires for Data Collection

Three standardized assessment instruments were employed to evaluate psychological and menopausal symptoms; DASS‐21, MKI, and VHI for the collection of data related to menopausal women. The final sets of eligible publications were then categorized based on AI paradigm, application to energy systems, engineering function, sustainability aim, and deployment consideration (see Table S2).

#### 2.4.1 Dass‐21

The DASS‐21 consists of three self‐report subscales, each with seven items designed to measure the negative emotional states of stress, anxiety, and depression. Scoring is performed by summing up the values for each subscale and multiplying the total by two. Based on the recommended scoring criteria, participants were categorised as follows:

- Depression: Normal (0–9), Mild (10–13), Moderate (14–20), Severe (21–27), Extremely Severe (≥ 28).
- Anxiety: Normal (0–7), Mild (8–9), Moderate (10–14), Severe (15–19), Extremely Severe (≥ 20)
- Stress: Normal (0–14), Mild (15–18), Moderate (19–25), Severe (26–33), Extremely Severe (≥ 34) (Bener et al. 2016).

#### 2.4.2 Mki

The MKI comprises 13 items, each rated on a 0–3 scale to reflect the severity of symptoms. The total score, ranging from 0 to 63, is determined by summing up all items with their respective weighting factors. Severity is classified as follows:

- None: 0–6
- Mild: 7–15
- Moderate: 16–30
- Severe: > 30 (Tao et al. 2013).

#### 2.4.3 Vhi

The VHI assessed participants' vaginal health by evaluating parameters such as elasticity, moisture, pH, mucosa, and discharge. Each factor was scored on a scale of 1–4, with lower scores indicating more severe vaginal symptoms. The researcher recorded these scores during a gynaecologic examination (R. Chen et al. 2018).

#### 2.4.4 Questionnaire

To assess the internal consistency reliability of the questionnaire‐based constructs used in the dataset, Cronbach's Alpha coefficients were calculated. The reliability analysis confirmed acceptable internal consistency for the psychological symptom measures derived from the DASS‐21 and menopausal symptom scores derived from the MKI and VHI instruments. These questionnaires are widely validated tools in clinical research and have demonstrated strong psychometric properties in previous studies.

### 2.5 Statistical Method

An independent samples *t*‐test is a statistical technique employed to assess whether there is a significant difference between the means of two independent groups. This method is often used when comparing the means from distinct populations or treatments in the case of case of this. A comprehensive explanation of how the independent *t*‐test was applied to determine which features exhibited significant differences after treatment is provided using hypotheses, *p*‐values etc.

#### 2.5.1 Hypotheses

The *t*‐test evaluates the following hypotheses

- Null hypothesis (H_0_): There is no significant difference between the means of HG and CG (*μ*_1_ = *μ*_2_).
- Alternative Hypothesis (H_1_): There is a significant difference between the means of HG and CG (*μ*_1_ ≠ *μ*_2_).

#### 2.5.2 Test Statistic

The test statistic *t* for an independent sample is calculated using the following Equation (1):

(1) $$t=\frac{{\bar{F}}_{b}-{\bar{F}}_{a}}{S_{p}\sqrt{\frac{1}{n1}+\frac{1}{n2}}}\;$$

where ${\bar{F}}_{b}$ and ${\bar{F}}_{a}$ means of the feature *F* of HG and CG. $\frac{1}{n1}$ and $\frac{1}{n2}$ are the sample sizes of the feature, *F* of HG and CG, respectively. $S_{p}$ is the pooled standard deviation mentioned in Equation (2):

(2) $$S_{p}=\sqrt{\frac{\left(n_{1}-1\right)S_{1}^{2}+\left(n_{2}-1\right)S_{2}^{2}}{n_{1}-\;n_{2}-2}}\;$$

where $S_{1}^{2}$ and $S_{2}^{2}$ are the variances of the feature *F* of HG and CG, respectively. The degrees of freedom, df, for the *t*‐test is given in Equation (3):

(3) $$\mathrm{df}=n_{1}\;-\;n_{2}-2$$

#### 2.5.3 *p*‐value

The *p*‐value is the probability of obtaining a test statistic at least as extreme as one observed, assuming that the null hypothesis is true. It is calculated based on the t‐distribution with the given degrees of freedom.

#### 2.5.4 Decision Rule

Significance Level (α): A predetermined threshold of 0.05 was used to decide whether to reject the null hypothesis.

- If the *p*‐value ≤ *α*, we reject the null hypothesis, indicating that there is a statistically significant difference in the feature, F, of HG and CG.
- If the *p*‐value > *α*, we fail to reject the null hypothesis, indicating that there is no statistically significant difference in the feature, F, of HG and CG.

### 2.6 Ensemble‐Based ML Models

The experimental data were analyzed in real‐time for drug classification using ensemble‐based ML models such as RF, GB, and AB. *k*‐fold CV with varying numbers of folds and random sampling with different numbers of training samples is one of the experimental setups employed. Under this random sampling, the dataset was divided into training and testing sets in proportions of 60%, 70%, 80%, and 90% for training data; the rest is used for testing. Each split was repeated multiple times to mitigate the effects of sampling bias and ensure robustness in performance evaluation. The evaluation for data were performed using a CV with *k*‐fold. In every fold, when a certain fold was treated as the validation set, then the other *k*‐1 folds were used to train. This was repeated k times, and the results from each fold were averaged to form the overall performance metrics. As a result, the overfitting was reduced, thus leading to better generalizability.

In this study, the ML task was formulated as a binary classification problem. To prevent target leakage, intervention group assignment was used exclusively for constructing the binary class labels and was excluded entirely from the predictor matrix. Consequently, the classifiers learned to distinguish intervention‐related clinical and questionnaire‐based outcome patterns solely from the observed predictor variables and did not have access to treatment group information during model training or validation. The outcome label represented the treatment group assignment, distinguishing participants receiving *P. anisum* (anise) intervention from those in the CG. The classification labels were derived from questionnaire‐based clinical features related to menopausal symptoms and psychological outcomes collected during the clinical study. The prediction was performed using post‐intervention measurements, where the ML models aimed to identify patterns associated with treatment response and symptom improvement. The classification results therefore represent the model's ability to distinguish the intervention group from the CG based on the observed clinical feature patterns.

The extracted information and data for each of the eligible studies were organized within a standardized analytical framework that included AI technique, energy‐system application, input/output data requirements, engineering function, performance measures, computational characteristics, interpretation, scalability, sustainability implications, and deployment considerations. The detailed data‐extraction matrix and supporting study‐level information are provided in Table S1.

#### 2.6.1 ML Validation Strategy

Due to the relatively small sample size (*N* = 60), a robust validation strategy was required to reduce sensitivity to random train–test splits. Therefore, model evaluation was performed using repeated stratified *k*‐fold CV, which preserves class distribution within each fold while providing multiple independent performance estimates. The final performance metrics were calculated as the mean ± standard deviation across CV folds, providing a more stable estimate of model generalization performance.

To provide an interpretable conventional benchmark, a logistic regression (LR) classifier was implemented as a baseline model and evaluated using the same preprocessing and repeated stratified CV protocol as the ensemble classifiers. The purpose of the LR model was not to serve as the primary analytical approach but to provide a transparent reference for assessing whether the ensemble methods offered improved classification performance. Performance metrics for the LR baseline, including sensitivity, specificity, accuracy, precision, F1‐score, and AUC, were therefore compared directly with those of RF, GB, and AB.

#### 2.6.2 ML Pipeline and Reproducibility

To ensure reproducibility and methodological transparency, the ML workflow was implemented using a structured pipeline that included data preprocessing, feature selection, model training, and evaluation within the CV framework.

1. *Feature set*: The final predictor set consisted of demographic, clinical, questionnaire‐derived, and relevant laboratory variables collected during the study. The intervention group assignment was explicitly excluded from the predictor matrix because it was used solely as the binary classification target. Feature selection was performed within the training data of each CV fold to minimize the risk of information leakage.
2. *Label construction*: The classification task was formulated as a binary outcome representing intervention group assignment (*Pimpinella anisum* treatment versus control). The intervention group variable was used exclusively for label construction and was not provided to the classifiers as an input feature. This separation between predictors and the target was maintained throughout preprocessing, model training, and validation.
3. *Data preprocessing*: Continuous variables were standardized using z‐score normalization, while categorical variables were encoded using one‐hot encoding where applicable. Importantly, preprocessing operations were applied within the training folds of the CV pipeline, and the resulting transformations were then applied to the corresponding validation data to prevent data leakage.
4. *Validation protocol*: Model evaluation was conducted using stratified *k*‐fold CV, ensuring that class distributions were preserved in each fold. To improve robustness, the procedure was repeated multiple times using fixed random seeds, enabling reproducible model training and evaluation.
5. *Hyperparameter optimization*: Model hyperparameters were optimized using a grid search procedure within the training folds. The search space included parameters such as regularization strength, learning rate, and tree depth (for tree‐based models). The final hyperparameter configuration was selected based on the highest average CV performance.

To ensure reproducibility, the ML pipeline was implemented with detailed specifications: hyperparameters for each classifier were optimized via grid search within the training folds (RF: number of trees = 100–500, max depth = 3–10; GB: learning rate = 0.01–0.2, n_estimators = 50–200; AB: n_estimators = 50–200, learning rate = 0.5–1.5). Repeated stratified *k*‐fold CV was applied, with five repetitions for stability. Fixed random seeds (42) were used across all experiments. All analyzes were performed using Python 3.10 with scikit‐learn 1.2.2 and NumPy 1.25.0.

Hyperparameter optimization was performed within the CV framework using grid search procedures as shown in Table 1. For the RF classifier, the search space included 100–500 trees and maximum tree depths of 3–10. For GB, learning rates ranging from 0.01 to 0.2 and 50–200 estimators were evaluated. For AB, the search included 50–200 estimators and learning rates ranging from 0.5 to 1.5. Repeated stratified *k*‐fold CV with five repetitions was applied to improve the stability of the performance estimates, and a fixed random seed of 42 was used across experiments. All analyzes were performed using Python 3.10, scikit‐learn version 1.2.2, and NumPy version 1.25.0.

**TABLE 1:** Final hyperparameter configurations of the evaluated classifiers.

Classifier	Hyperparameter search range reported in manuscript
Random forest	Number of trees = **100–500**; maximum depth = **3–10**
Gradient boosting	Learning rate = **0.01–0.2**; n_estimators = **50–200**
AdaBoost	n_estimators = **50–200**; learning rate = **0.5–1.5**

Hyperparameters were selected using the tuning procedure described in Section 2.6.2. The reported values represent the final selected configurations used for the corresponding model evaluation.

#### 2.6.3 ML‐Based Proposed Algorithms

The performance measures include sensitivity, specificity, accuracy, F1, precision, and area under the curve (AUC). To ensure optimal performance, hyperparameter tuning using grid search and random search methods was carried out for each classifier. In this manner, the classifiers were rigorously evaluated under diverse data distributions and experimental setups, considering the valuable performance insights facilitated by random sampling and CV. We calculated the performance measures by Equations (4)–(8), and the pseudocode is shown in Algorithms 1, 2, 3:

(4) $$\mathrm{Sensitivity}\;=\frac{\mathrm{TP}}{\mathrm{TP}\;+\mathrm{FN}}$$

(5) $$\mathrm{Specificity}=\frac{\mathrm{TN}}{\mathrm{TN}+\mathrm{FP}}$$

(6) $$\mathrm{Accuracy}=\frac{\mathrm{TP}+\mathrm{TN}}{\mathrm{FP}+\mathrm{FN}+\mathrm{TN}+\mathrm{TP}}$$

(7) $$F1=\frac{\mathrm{TP}}{\mathrm{TP}\;+\frac{1}{2}\;\left(\mathrm{FP}+\mathrm{FN}\right)}$$

(8) $$\mathrm{Precision}=\frac{\mathrm{TP}}{\mathrm{TP}+\mathrm{FP}}$$

where TP denotes the number of true positives, TN denotes the number of true negatives, FP denotes the number of false positives, and FN denotes the number of false negatives.

ALGORITHM 1 **Pseudocode of the RF algorithm.**

**Table:** 

1. ***Input***: Dataset D, number of trees T, number of features F for splitting
2. ***Output***: Ensemble model *M*_RF_
3. ***Procedure***:
For i=1 to T:
(i) Draw a bootstrap sample *D_i_* from the dataset *D* .
(ii) Build a decision tree *T_i_* using *D_i_* :
At each node, randomly select F features.
Split the node using the feature and threshold that maximizes information gain or minimizes impurity.
Repeat until stopping criteria are met.
End For
4. ***Combine*** *Predictions of all T Trees*:
For classification: Use majority voting.
For regression: Use the average of predictions.
5. ***Return***: Ensemble model M_RF_

ALGORITHM 2 **Pseudocode of the GB algorithm.**

**Table:** 

1. ***Input***: Dataset *D*, number of iterations *N*, learning rate *η*, base model h
2. ***Output***: Ensemble model *M*_GB_
3. ***Procedure***:
Initialize the model with a constant predictor:
Initialize the model with a constant predictor: $F_{0}\;\left(x\right)=$ $\mathrm{argmi}n_{\gamma }\sum \mathrm{Loss}\left(y,\gamma \right).$
For i=1 to N:
(i) Compute the negative gradient (pseudo‐residuals)
$\;r_{i}=-\left[\frac{\partial \mathrm{Loss}(y,F_{i-1}\left(x\right))}{\partial F_{i-1}\left(x\right)}\right].$
(ii) Fit a weak learner $h_{i}\left(x\right)$ to the pseudo‐residuals $r_{i}.$
(iii) Update the model:
$F_{i}\;\left(x\right)=F_{i-1}\;\left(x\right)+\eta \cdot h_{i}\left(x\right).$
End For
4. ***Combine the weak learners***: $M_{\mathrm{GB}}\;\left(x\right)=F_{N}\;\left(x\right).$
5. ***Return***: Ensemble model *M*_GB_

Figure 3 illustrates the process of building an RF model. It begins with a large dataset (*D*), which is then used to generate multiple bootstrap samples (D1, D2, …, DT). For each of these samples, a decision tree (T_i) is constructed, with splits based on criteria like “Max Depth” or “Min Samples.” This process is repeated for several trees (T), and the individual decisions from each tree are combined to form a collective prediction. Finally, the ensemble model is created by aggregating the results from all the trees, producing a robust final prediction (MRF). This method enhances model accuracy by leveraging the diversity of multiple decision trees.

**FIGURE 3:**
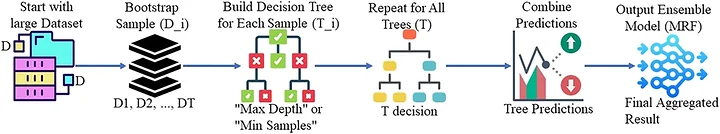
Designing an ensemble‐based RF algorithm.

**FIGURE 4:**
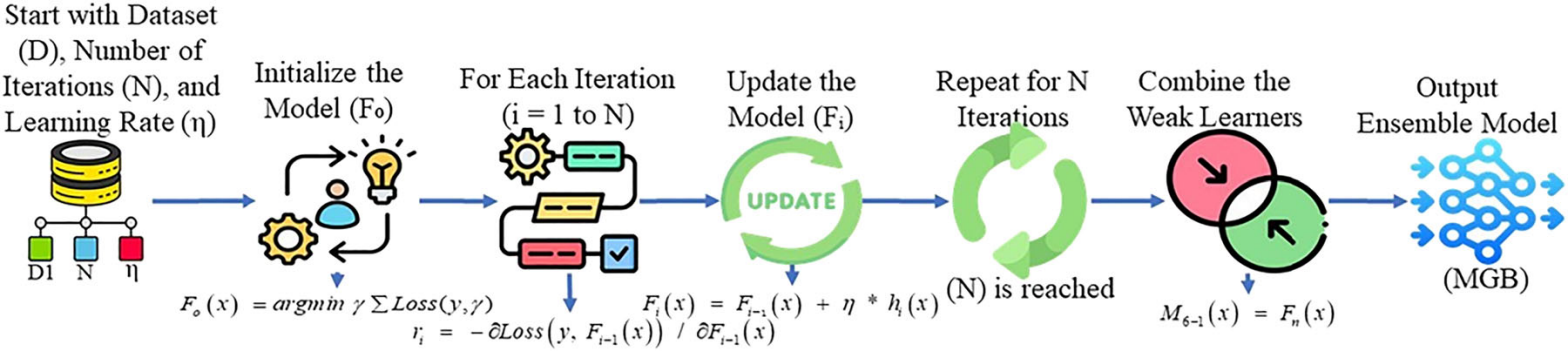
Designing an ensemble‐based GB algorithm.

**FIGURE 5:**
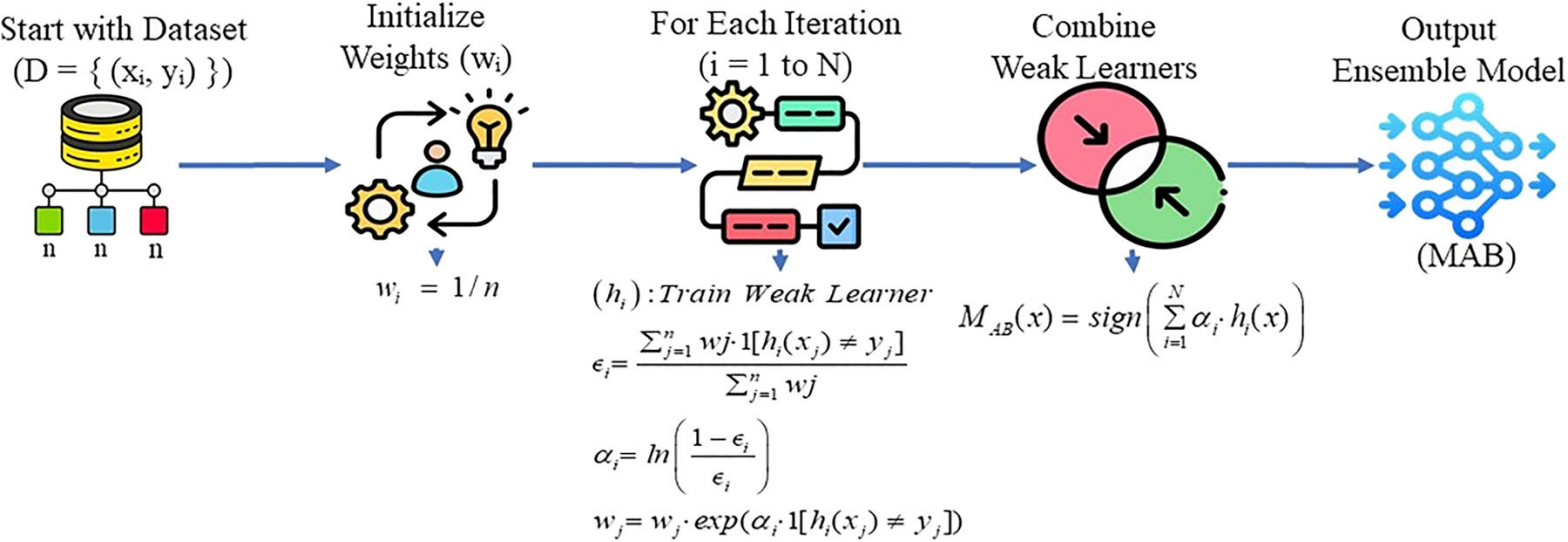
Designing an ensemble‐based AB algorithm.

Figure 4 describes the process of building an ensemble model using a technique similar to boosting. It starts with a dataset (*D*), along with the number of iterations (*N*) and a learning rate (*η*). The model (*F*_0_) is initialised, and then for each iteration (from 1 to *N*), the model is updated based on the loss function. In each iteration, the model is updated by adding a weighted term (*η* × *h*(*x*)), where *h*(*x*) is the weak learner, and the learning rate controls the contribution of the learner to the final model. This process is repeated for N iterations, combining the weak learners into a final ensemble model (MGB). The result is a more accurate model created by combining multiple weak learners, which improves performance through iterative updates.

ALGORITHM 3 **Pseudocode of the AB algorithm.**

**Table:** 

1. ***Input***: Dataset $D={\left\{\left(x_{i},y_{i}\right)\right\}}_{i=1}^{n}$, number of iterations N, weak learner h
2. ***Output***: Ensemble model *M*_AB_
3. ***Procedure***:
Initialize weights $w_{i}=\frac{1}{n}$ for all i=1,…,n.
For i=1 to N:
(i) Train a weak learner $h_{i}\left(x\right)$ using the weighted dataset D.
(ii) Calculate the weighted error:
$\;\varepsilon_{i}=\frac{\sum_{j=1}^{n}w_{j}\cdot 1\left[h_{i}\left(x_{j}\right)\neq y_{j}\right]}{\sum_{j=1}^{n}w_{j}}\;.$
(iii) Compute the model weight:
$\;\alpha_{i}=\;\ln\left(\frac{1-\varepsilon_{i}}{\varepsilon_{i}}\right).$
(iv) Update instance weights:
$\;w_{j}=w_{j}\;\cdot \exp\left(\alpha_{i}\cdot 1\left[h_{i}\left(x_{j}\right)\neq y_{j}\right]\right)$ for all j.
Normalize $w_{j}$ to ensure $\sum \;w_{j}=1.$
End For
4. ***Combine the weak learners***:
$M_{\mathrm{AB}}\;\left(x\right)=\;\mathrm{sign}\left(\sum_{i=1}^{N}\alpha_{i}\cdot h_{i}\left(x\right)\right).$
4. ***Return***: Ensemble model MAB

Figure 5 illustrates the process of building an ensemble model using a method similar to AB. It begins with a dataset (*D*) and initializes weights (*w_i_*) for each data point, with an initial weight of 1/*n* for all points. Then, for each iteration (from 1 to *N*), a weak learner (*h_i_*) is trained, and the weights are updated based on the performance of the weak learner. The error rate (*ϵ_i_*) of the learner is calculated, and the learner's weight (*α_i_*) is adjusted accordingly, giving more weight to misclassified points. After N iterations, the weak learners are combined into an ensemble model (MAB), where the final prediction is made by taking the weighted majority vote of all weak learners. The result is an ensemble model that improves performance by focusing on difficult‐to‐classify instances in each iteration.

### 2.7 ML Feature Timepoints and Selection Strategy

The machine‐learning models in this study were trained using clinical and questionnaire‐derived variables collected at baseline and post‐intervention follow‐up assessments (weeks 8 and 16). Because the feature set includes measurements obtained after the intervention, the resulting models should be interpreted as classifiers of intervention‐related clinical and psychological outcome patterns rather than prospective prediction models. Accordingly, the analysis evaluates patterns associated with treatment response and observed group differences and does not estimate an individual's future risk of developing psychological disorders.

To reduce the risk of information leakage and optimistic bias, feature selection was performed within the training folds of the CV procedure, ensuring that no information from the validation data were used during model training. Future studies should develop and validate baseline‐only models in independent prospective cohorts to evaluate whether early‐risk prediction or screening of menopausal psychological symptoms is feasible.

## 3 Results and Discussion

### 3.1 Experimental Results

The menopausal women with psychological symptoms were in the age group of 40–60 years. The mean age in the HG and CG was 44.50 ± 3.911 and 46.90 ± 5.54, respectively (no statistically significant difference at baseline). The BMI of HG and CG was 30.17 ± 5.29 and 27.82 ± 4.76 kg/m^2^ (*p* = 0.08, unpaired *t*‐test), respectively. The majority of participants in HG and CG had only a primary school education (13 [43.33%] vs. 7 [23.33%], *p* = 0.35; test used: Chi‐square test), and 60% in the HG and 63.33% in the CG had a middle school certificate or higher education. In terms of menopausal status, 63.33% and 53.33% of participants were in the menopausal transition stage, while 36.67% and 46.67% were in the post‐menopausal stage in the HG and CG, respectively. The sociodemographic variables were very similar at baseline. For improved interpretability, the machine‐learning workflow and performance visualizations were formatted with enlarged labels, legends, and annotations and with increased spacing between graphical elements to ensure readability at publication scale.

At baseline, no significant differences were observed between the two groups across DASS‐21 domains, confirming comparability. However, by the 8 week, the HG exhibited a substantial effect size across all three domains, while the CG's changes remained statistically insignificant within the group. At 8 weeks, participants in the HG demonstrated a significantly greater reduction in mean scores for depression, anxiety, and stress on the DASS‐21 scale compared to the CG (*p* < 0.001). For depression, the HG showed a mean reduction from 22.00 ± 5.35 (95% CI: 20.01–23.98) at baseline to 3.47 ± 5.43 (95% CI: 1.43–5.49) at 8 weeks, whereas the CG exhibited a lesser reduction from 19.47 ± 5.36 (95% CI: 17.4–21.46) to 14.20 ± 7.27 (95% CI: 11.48–16.91). The between‐group comparison at 8 weeks was statistically significant (*p* < 0.001). About anxiety, the HG demonstrated a decrease from 30.40 ± 5.08 (95% CI: 28.50–32.29) at baseline to 4.73 ± 8.29 (95% CI: 1.63–7.83) at 8 weeks. In contrast, the CG showed a minor reduction from 27.93 ± 5.91 (95% CI: 25.72–30.13) to 21.73 ± 10.49 (95% CI: 17.81–25.64), with a significant difference between groups (*p* < 0.001). For stress, the HG's mean score reduced from 28.67 ± 4.15 (95% CI: 27.11–30.21) to 5.87 ± 7.96 (95% CI: 2.89–8.84), while the CG showed a smaller change from 27.67 ± 5.46 (95% CI: 25.62–29.70) to 21.67 ± 10.45 (95% CI: 17.76–25.56). The difference between groups was again statistically significant (*p* < 0.001). However, the HG showed a statistically significant difference in comparison with the CG at first, second, third, and fourth months (*p* < 0.001), respectively.

At baseline, the MKI mean scores were 37.53 ± 3.86 (95% CI: 36.09–38.97) in the HG and 35.70 ± 4.04 (95% CI: 34.19–37.21) in the CG, with no statistically significant difference between the groups (*p* > 0.05, Wilcoxon Matched Paired Test). However, significant differences were observed from the first month onward. At Week 4, the HG exhibited a substantial reduction in MKI scores to 17.10 ± 6.13 (95% CI: 14.81–19.39), compared to 28.23 ± 7.08 (95% CI: 25.58–30.87) in the CG, with a mean difference of 11.13 (95% CI: −2.266 to −1.084, *p* < 0.0001). This trend continued at Week 8, where the HG score further declined to 6.53 ± 11.71 (95% CI: 2.16–10.96), while the CG remained elevated at 28.17 ± 10.84 (95% CI: 24.11–32.21), yielding a significant mean difference of 21.64 (95% CI: −2.525 to −1.297, *p* < 0.0001). Similarly, at Week 12, the HG maintained a lower MKI score of 6.37 ± 12.03 (95% CI: 1.87–10.85), compared to 29.43 ± 10.30 (95% CI: 25.58–33.28) in the CG, with a mean difference of 13.06 (95% CI: ‐2.683 to ‐1.424, *p* < 0.0001). By Week 16, the HG showed a slight increase to 12.60 ± 10.60 (95% CI: 8.64–16.55), while the CG remained high at 30.63 ± 7.46 (95% CI: 27.84–33.42), resulting in a significant mean difference of 18.03 (95% CI: −2.581 to −1.342, *p* < 0.0001). For the VHI, no significant difference was noted at baseline between HG (8.9 ± 2.05, 95% CI: 8.13–9.67) and CG (8.8 ± 1.60, 95% CI: 8.2–9.4) (*p* = 0.83). However, by Week 8, the HG showed a marked improvement to 13.33 ± 2.26 (95% CI: 12.48–14.17) compared to 9.32 ± 2.44 (95% CI: 8.42–10.24) in the CG, with a highly significant difference (*p* < 0.0001). No side effects were reported in both groups. All biochemical biomarkers were within normal limits.

Flavonoids are recognized for their antidepressant and anxiolytic properties (Mosavat et al. 2019). Extracts of ethanol (70%) and aqueous solutions demonstrated antidepressant activity in both animal and human studies (Mahboubi and Mahboubi 2021; Bährle‐Rapp 2007). The antidepressant activity is attributed to antioxidant compounds, including anethole, which potentially inhibit the reuptake of serotonin (5‐HT) and monoamine oxidase; thereby, 5‐HT levels in synaptic clefts are increased, and 5‐HT receptors are sensitized. This mechanism contributes to the antidepressant effects. Besides, bioactive plant metabolites like quercetin, caffeic acid, and catechins have demonstrated antidepressant and anxiolytic properties in previous studies. Quercetin's effects involve reducing the excitotoxicity and enhancing serotonin levels, while caffeic acid's anxiolytic effects are associated with its action on N‐methyl‐d‐aspartate (NMDA) receptors. In immature females, oral administration of anethole (80 mg/kg) for 3 days significantly increased uterine weight compared to oestradiol valerate (Mahboubi and Mahboubi 2021). Phytoestrogens play a key role in mitigating hot flashes by helping to balance estrogen levels in the body. Hot flashes are often associated with the hormonal changes, and phytoestrogens help to regulate the estrogen levels. Depression precedes the hot flashes, which are linked to the fluctuations in reproductive hormone levels.

The selective action of isoflavones, such as genistein and daidzein, on the target tissues is attributed to the differential localization of estrogen receptors (ERs). Genistein and daidzein bind to estrogen receptors with affinities 100 and 1000 times lower than 17β‐oestradiol, respectively. They preferentially bind to the estrogen receptor, β (ERβ), over estrogen receptor, *α* (ER*α*). Bone, brain, bladder, and blood vessels are rich in ERβ, while breast, uterus, and ovary express both ER*α* and ERβ, explaining tissue‐specific effects. Phytoestrogens exhibit antiestrogenic effects by inhibiting enzymes like aromatase, 17β‐HSD, and 3β‐HSD, regulating estrogen metabolism and aiding hormonal balance therapy (Pampaloni et al. 2009).

### 3.2 Statistical Results

In this study, an independent two‐sample *t*‐test was employed to determine the features exhibiting statistical significance that differed between the HG and CG. The features were selected based on their *p*‐values, with a threshold of *p* ≤ 0.05, which signifies a meaningful difference in mean values between the two groups. This method of feature selection emphasizes those variables which are most likely to be affected by the experimental intervention. Also, Diagnostic analysis showed no evidence of problematic multicollinearity among predictors, with all VIF values remaining within acceptable thresholds.

The key features identified through this approach include both biological and demographic variables. Eosinophils BT (*p* = 0.0042) and monocytes BT (*p* = 0.0103) showed significant differences, indicating potential changes in immune markers. Additionally, time‐dependent variables like MKI‐12 WK (*p* = 6.7 × 10^−11^), Q1‐8 WK (*p* = 2.6 × 10^−10^), and several others at 8‐week and 16‐week intervals exhibited extremely low *p*‐values, reflecting substantial intervention effects over time.

The selected features span biological, temporal, and demographic dimensions and provide important insights into how the experimental treatment impacts health and psychological outcomes over time. Focusing on the features (which are changed statistically significantly) usually ensures that the analysis remains centered on the most influential variables, thereby enhancing the overall precision and relevance of the findings. Figure 6 demonstrates the *p*‐values of the selected features using an independent samples *t*‐test.

**FIGURE 6:**
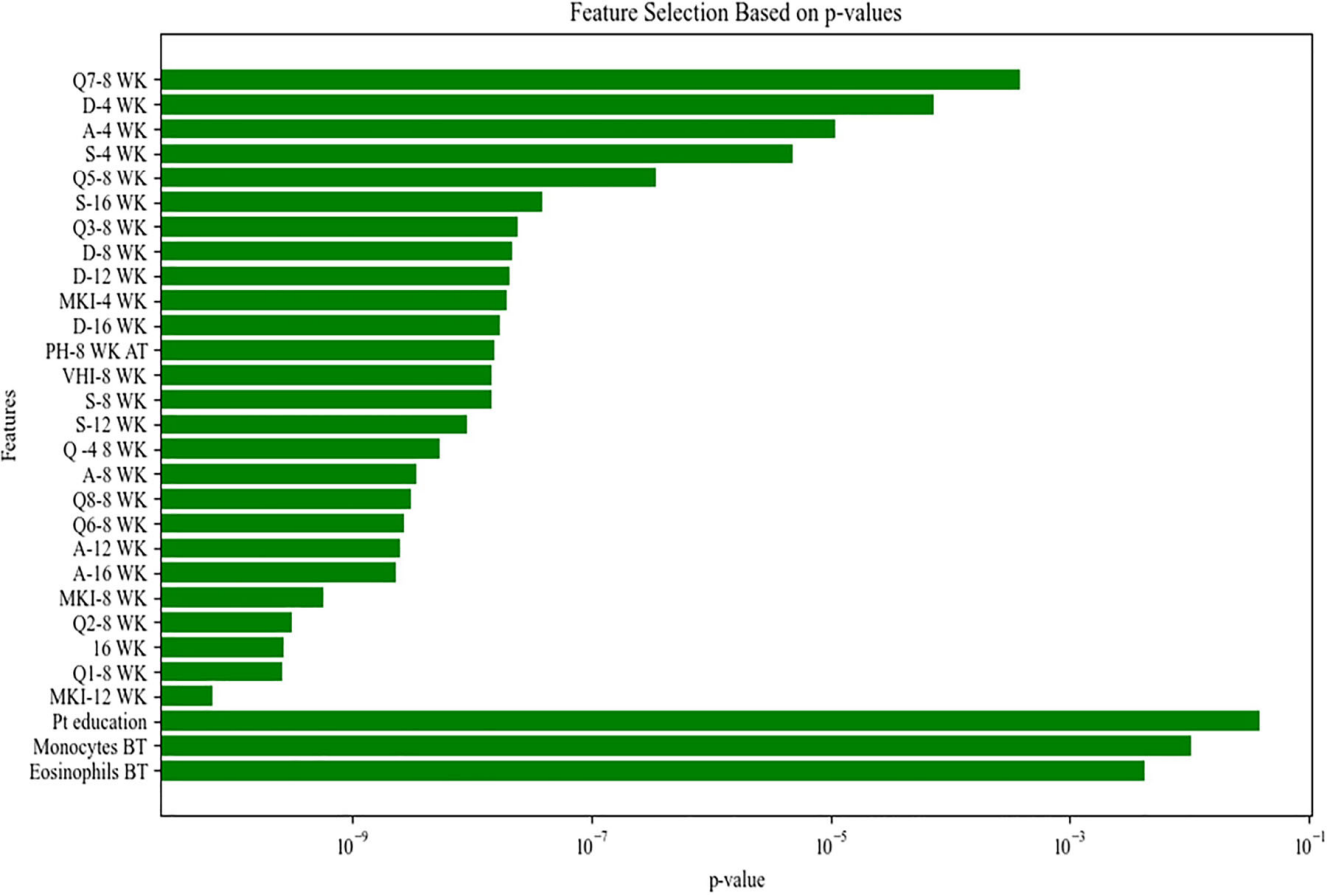
*p*‐values of the selected features using an independent samples *t*‐test. Abbreviation: A, anxiety; D, depression; MKI, modified Kupperman index; S, stress; VHI, vaginal health index; WK, week.

### 3.3 Classification Results

The comparative CV results are presented in Table 2. The methodological trade‐offs that need to be taken into account when choosing an AI approach for a given sustainable energy application are discussed (see Table S3 for the extended comparison). Among the ensemble classifiers, the RF model demonstrated the strongest and most consistent overall performance. RF achieved accuracies of 0.867, 0.917, 0.917, and 0.883 under the CV‐2, CV‐3, CV‐5, and CV‐10 validation settings, respectively. Its AUC values remained high across all validation settings, ranging from 0.939 to 0.957. The highest RF sensitivity was 0.933 under CV‐3, while the highest specificity was 0.967 under CV‐5. The model also achieved its highest precision of 0.963 under CV‐5. GB demonstrated competitive performance, particularly under CV‐5, where it achieved a sensitivity of 0.933, an accuracy of 0.900, and an AUC of 0.914. AB showed comparatively lower accuracy across the evaluated validation settings, ranging from 0.767 to 0.833. Overall, the results indicate that RF provided the most robust classification performance among the three ensemble classifiers evaluated in this study.

**TABLE 2:** Performance of the system based on ensemble learning techniques using random sampling models.

Classifier		Model	Training size	Sensitivity	Specificity	Accuracy	F1	Precision	AUC
RF		CV‐2	60%	0.867	0.867	0.867	0.867	0.867	0.857
GB		CV‐2		0.800	0.733	0.767	0.774	0.750	0.833
AB		CV‐2		0.800	0.833	0.817	0.814	0.828	0.817
RF		CV‐3	70%	**0.933**	0.900	**0.917**	**0.918**	0.903	**0.940**
GB		CV‐3		0.867	0.800	0.833	0.839	0.812	0.852
AB		CV‐3		0.800	0.733	0.767	0.774	0.750	0.767
RF		CV‐5	80%	0.867	**0.967**	**0.917**	**0.912**	0.963	**0.939**
GB		CV‐5		**0.933**	0.867	0.900	**0.903**	0.875	0.914
AB		CV‐5		0.800	0.833	0.817	0.814	0.828	0.817
RF		CV‐10	90%	0.833	**0.933**	**0.883**	**0.877**	**0.926**	**0.944**
GB		CV‐10		**0.900**	0.800	0.850	**0.857**	0.818	0.917
AB		CV‐10		0.800	0.867	0.833	0.828	0.857	0.833
	Mean			0.850	0.844	0.847	0.848	0.047	0.877
	±SD			0.049	0.068	0.049	0.047	0.061	0.061

Model performance was evaluated using accuracy, precision, recall, and F1‐score, reported as mean ± standard deviation across CV folds. Confusion matrices were generated to visualize class‐level prediction performance, and receiver operating characteristic (ROC) curves were used to assess discriminative ability. These analyzes provide a more comprehensive interpretation of model performance given the limited sample size. To enhance interpretability, the feature importance from the RF model was examined. The most influential features contributing to the model's predictions included MKI‐12 WK, Q1‐8 WK, and immune biomarkers such as eosinophils BT and monocytes BT. These features align with clinical observations of symptom improvement and immune response during the intervention period, thereby providing insights into key factors driving the model's decision‐making.

Figure 7 provides line plots that show the performance of the classifiers at various training sizes (60%, 70%, 80%, and 90%). The RF classifier consistently outperforms the other classifiers in all metrics (sensitivity, specificity, accuracy, F1, precision, and AUC) at each training size. The performance of GB and AB, while still notable, drops significantly as training size increases, especially in metrics like AUC, where RF maintains its superior score across all training sizes. These plots emphasize that RF is the most reliable classifier for this problem, demonstrating robust performance as the training size increases.

**FIGURE 7:**
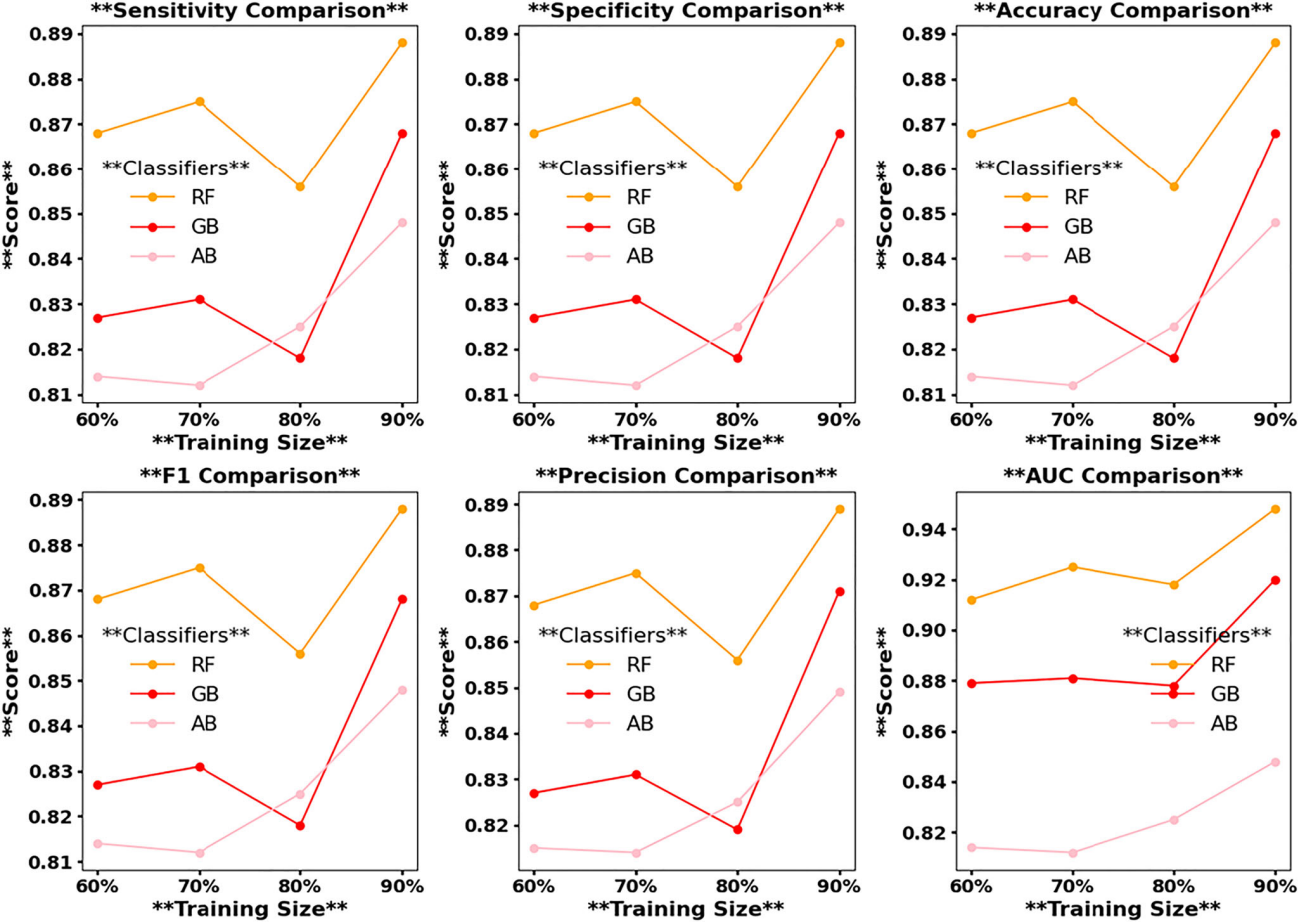
ROC results of the ensemble learning techniques using random sampling.

Figure 8 compares the performance of three classifiers, RF, GB, and AB, across various metrics with different training sizes. The results show that RF consistently outperforms both GB and AB in all metrics. As the training size increases, RF's performance improves significantly, particularly in AUC and accuracy. GB shows steady improvements but lags behind RF, especially in Precision and AUC. AB consistently exhibits the lowest scores across all training sizes and metrics, indicating its lesser effectiveness in comparison to RF and GB. The bolded titles and axis labels emphasize the classifier comparisons and the impact of increasing training size on model performance. The performance metrics shown in Table 3 are the classifiers across the different CV models. The RF classifier consistently achieves the best results across all metrics and model sizes, with a mean AUC of 0.877. GB and AB, while still effective, show comparatively lower performance, particularly in AUC. The table confirms the trend seen in the figures, with RF being the most reliable model in the classification of psychological disorders in menopausal women.

**FIGURE 8:**
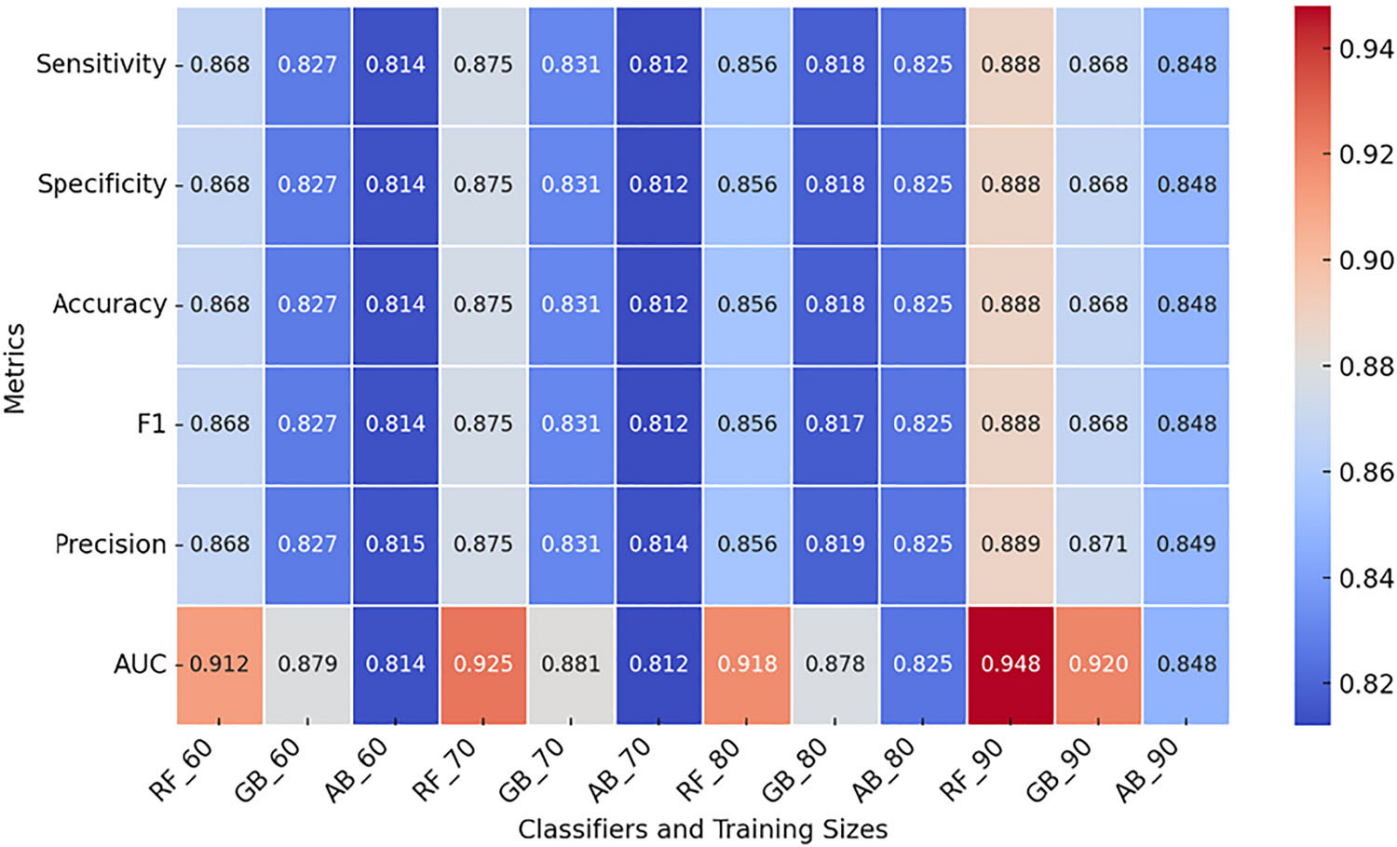
Heatmap of the ensemble‐based ML techniques using random sampling models.

**TABLE 3:** Performance of the system based on ensemble learning techniques using cross‐validation models.

Classifier	Model	Sensitivity	Specificity	Accuracy	F1	Precision	AUC
RF	CV‐2	0.867	0.867	0.867	0.867	0.867	0.957
GB		0.800	0.733	0.767	0.774	0.750	0.833
AB		0.800	0.833	0.817	0.814	0.828	0.817
RF	CV‐3	**0.933**	**0.900**	**0.917**	**0.918**	**0.903**	**0.940**
GB		0.867	0.800	0.833	0.839	0.812	0.852
AB		0.800	0.733	0.767	0.774	0.750	0.767
RF	CV‐5	0.867	0.967	0.917	0.912	0.963	0.939
GB		0.933	0.867	0.900	0.903	0.875	0.914
AB		0.800	0.833	0.817	0.814	0.828	0.817
RF	CV‐10	0.833	0.933	0.883	0.877	0.926	0.944
GB		0.900	0.800	0.850	0.857	0.818	0.917
AB		0.800	0.867	0.833	0.828	0.857	0.833
Mean		0.850	0.844	0.847	0.848	0.848	0.877
±SD		0.049	0.068	0.049	0.047	0.061	0.061

Figure 9 presents a comparison of three classifiers, such as RF, GB, and AB, across various performance metrics. The results are displayed for different model sizes (CV‐2, CV‐3, CV‐5, and CV‐10). Overall, the RF classifier demonstrated the strongest performance among the evaluated models for distinguishing intervention‐related clinical and psychological outcome patterns in menopausal women. These findings indicate that ensemble learning techniques can effectively analyze the multidimensional clinical and questionnaire‐based patterns observed during the intervention study. The classification performance should, however, be interpreted within the context of the available follow‐up measurements and should not be considered evidence of prospective prediction of psychological disorders.

**FIGURE 9:**
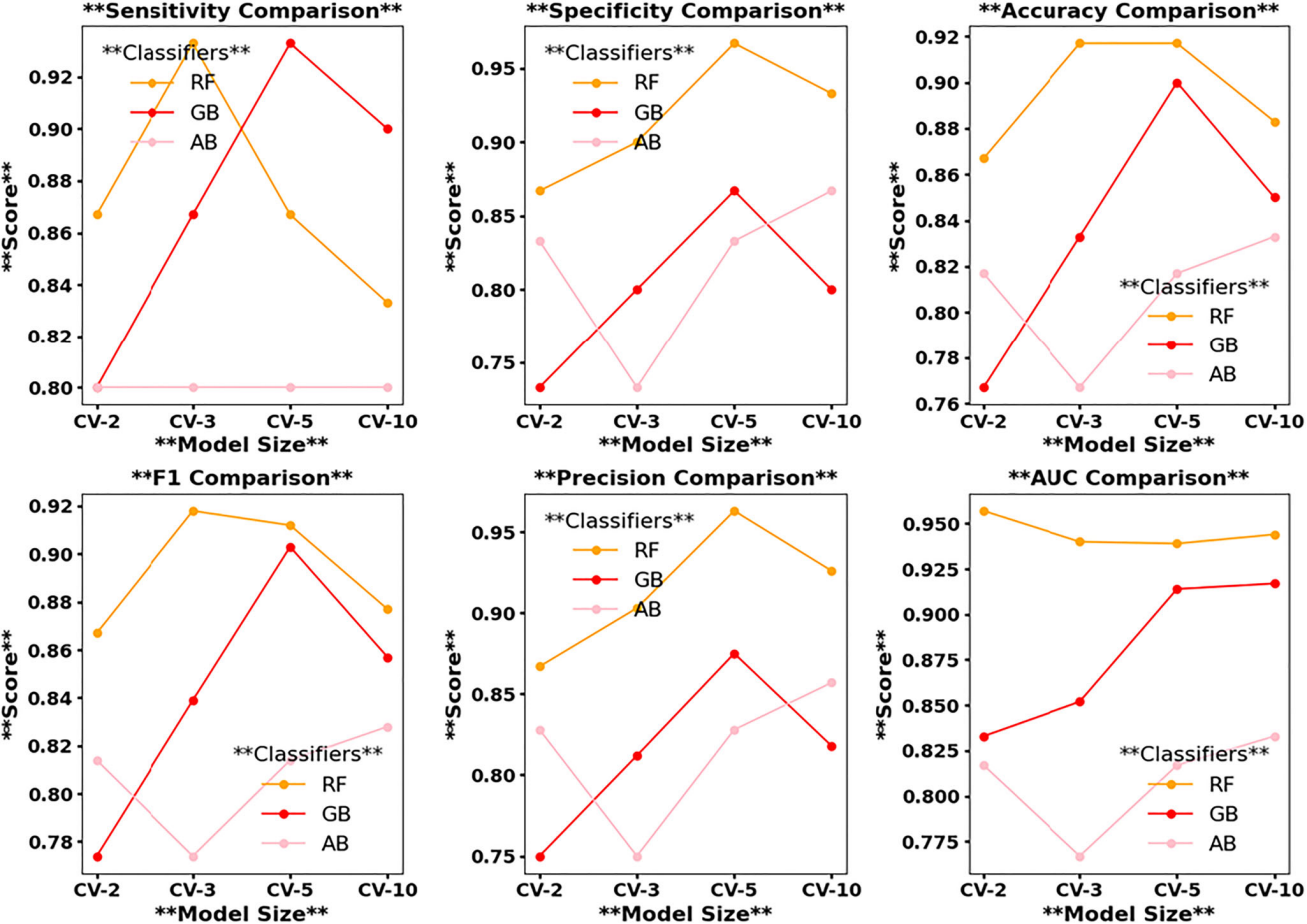
Comparison of the ensemble‐based ML techniques using CV models.

The heatmap in Figure 10 displays the performance metrics for each classifier across different model sizes. RF consistently outperforms GB and AB, with the highest AUC score of 0.957 at CV‐2, followed by high AUC values in the other model sizes. In general, RF shows superior performance in terms of all metrics, especially in AUC, where it reaches 0.944 at CV‐10. The line plots further illustrate these results, showing that RF leads in all metrics across model sizes, while GB and AB lag behind, particularly in AUC and precision. The plots highlight RF's robust performance across various model sizes, making it the top performer for this classification task.

**FIGURE 10:**
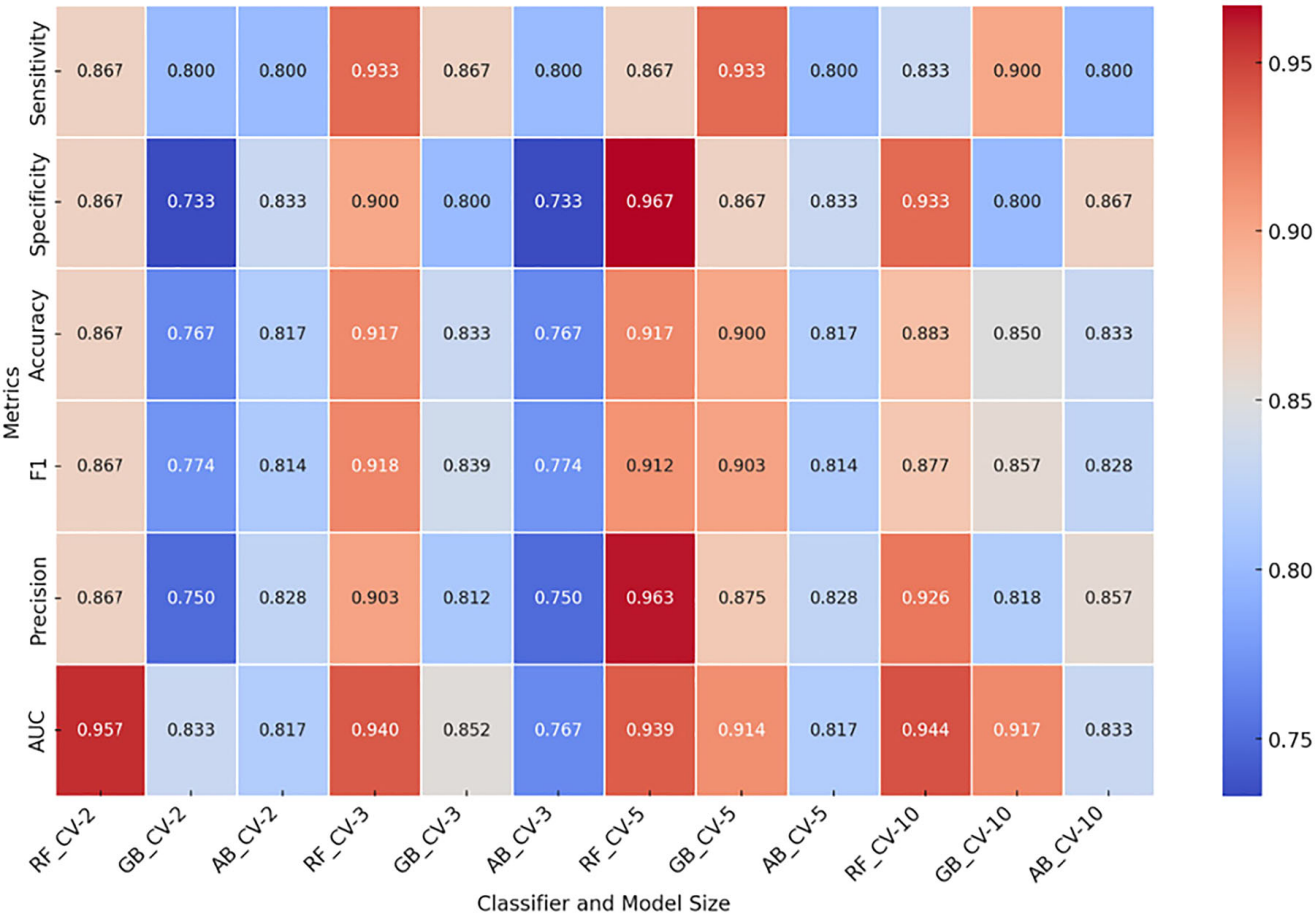
Heatmap of the ensemble‐based ML techniques using CV models.

### 3.4 Comparison With Previously Published Study

A previous study confirmed that sage extract significantly reduces the severity of various symptoms in postmenopausal women (Sheng et al. 2022). A study of 82 participants found that saffron led to more substantial improvements in mood and psychological symptoms compared to a placebo (Roozbeh et al. 2019). Fennel seed was also found to alleviate the menopausal symptoms significantly (Sun et al. 2019). Other herbal remedies such as *Tribulus terrestris* (Fatima and Sultana 2017), *Melissa officinalis* (Shirazi et al. 2021), and *Glycyrrhiza glabra* (Asgari et al. 2015; Nahidi et al. 2012) have also shown effectiveness in managing menopausal symptoms. A systematic review reported that lavender aromatherapy and oral capsules were effective for alleviating sleep disturbances, vasomotor symptoms, and psychological issues (Eatemadnia et al. 2019). Consistent with previous findings, this study observed reduced symptom severity among participants. Women in the HG also showed improved VHI and vaginal pH compared to the CG by the second month post‐intervention. A study demonstrated that modified Erzhi granules (MEG) significantly improved vulvovaginal atrophy (VVA) and other menopausal symptoms. Similarly, the current study showed the significant reductions in depression, anxiety, stress, and other menopausal symptoms, along with an improvement in the VHI score (Ghaffari et al. 2020; Lee et al. 2021).

Table 4 compares the accuracy achieved by different classifiers across several studies. The accuracy values range from 60.3% to 91.7%. The proposed study stands out with the highest accuracy (91.7%) in predicting psychological disorders using RF and herbal medicine. In contrast, the studies from 2022 to 2024 using classifiers like DT, AB, and NB show lower accuracy, reinforcing the superior performance of the proposed approach in handling psychological disorders in menopausal women. This highlights the effectiveness of using RF and herbal medicine in achieving high classification accuracy compared to traditional methods.

**TABLE 4:** Comparison between previous and proposed studies based on different interventions with ML algorithms.

Author	Year	Objective	Domain	Major	ML	Intervention	Acc. (%)
Pal et al. (2022)	2022	Monitoring	Anxiety	ECG, SAS	RF	MSY	80.00
Qayyum et al. (2023)	2023	Therapeutic efficacy	uPID	VAS, McPS	DT	USM	61.80
Fazmiya et al. (2024)	2024	Efficacy, classification	HMB	PBLAC	AB	USM	60.30
Sumbul et al. (2024)	2024	Efficacy, classification	uPID	VAS, SF‐12	NB	USM	68.30
Proposed study		Classification of intervention‐related outcome patterns	Psychological and menopausal outcome patterns	DASS‐21, MKI, VHI	RF	Herbal Medicine	91.70

Abbreviations: AB, AdaBoost; DT, decision tree; ECG, electrocardiogram; HMB, heavy menstrual bleeding; McPS, McCormack pain scale; MSY, mano shakti yoga; NB, naive bayes; PBLAC, pictorial blood loss assessment chart; RF, random forest; SAS, self‐rating anxiety scale; SF‐12, short form‐12; uPID, uncomplicated pelvic inflammatory disease; USM, unani system of medicine; VAS, visual analogue scale.

Figure 11 presents a comparative analysis between the proposed and previous studies related to female disorders. The bar graph shows the accuracy of these classifiers used in different research papers from 2022 to 2024, with the proposed study achieving the highest accuracy of 91.7%. In comparison, previous studies report lower accuracy rates, such as 80% for RF in anxiety monitoring, 61.8% for DT in therapeutic efficacy, and 60.3% for AB in classification tasks.

**FIGURE 11:**
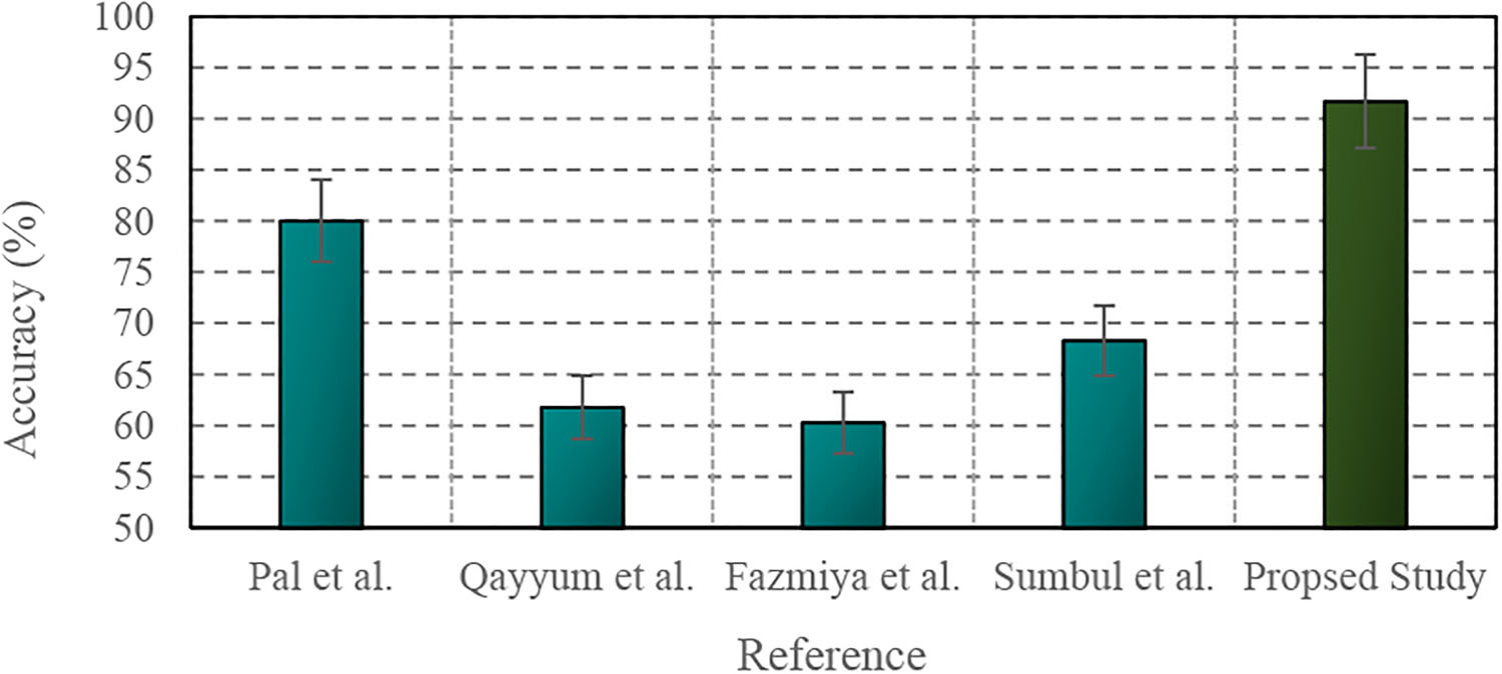
Accuracy comparison for existing classifiers of different authors and this proposed study.

### 3.5 Strengths and Applications of the Study

The study's uniqueness lies in its inclusive assessment of *P. anisum's* efficacy and safety in addressing depression, anxiety, stress, menopausal symptoms, and vaginal health in menopausal women. Using validated tools like DASS‐21, MKI, and vaginal health assessments strengthens the study's findings. The randomized, double‐blinded, herbal‐controlled design, coupled with high participant compliance and minimal loss to follow‐up, further enhances the study's credibility. The comparative evaluation demonstrated that the RF classifier achieved the strongest and most consistent performance among the three ensemble models evaluated. Across the different CV settings, RF achieved accuracies ranging from 0.867 to 0.917 and AUC values ranging from 0.939 to 0.957. In comparison, GB achieved accuracies ranging from 0.767 to 0.900 and AUC values ranging from 0.833 to 0.917, while AB achieved accuracies ranging from 0.767 to 0.833 and AUC values ranging from 0.767 to 0.833. These findings indicate that RF provided the most robust discrimination among the evaluated ensemble approaches, particularly when considering accuracy, specificity, precision, and AUC across multiple CV settings.

The application of this study lies in its potential to offer a natural, safe, and effective treatment option for managing menopausal symptoms, including depression, anxiety, stress, and vaginal health issues. The findings could be used to support the integration of *P. anisum* into clinical practice as a complementary remedy for menopausal women, providing an alternative to conventional therapies. In addition, this study can potentially be deployed as a web‐based screening tool to assist clinicians in the early identification of psychological disorders in menopausal women. A prototype interface integrated with self‐reported symptom entry can be designed to facilitate real‐time decision support in a community setting.

The machine‐learning component of this study provides an analytical framework for examining intervention‐related clinical and psychological outcome patterns. Rather than serving as a prospective screening or early‐risk prediction system, the models are intended to support the analysis and interpretation of multidimensional treatment‐response patterns observed during the intervention study. Such approaches may assist researchers and clinicians in evaluating changes across psychological and menopausal symptom measures and in exploring factors associated with differential treatment responses. The present findings should not be interpreted as demonstrating the ability to identify psychological disorders before their occurrence or to estimate future individual risk. Because post‐intervention measurements were included in the feature set, prospective screening applications cannot be established from the current analysis. Future studies using baseline‐only variables, larger cohorts, external validation, and prospectively defined outcomes are required before developing ML‐based early screening or risk prediction tools for menopausal psychological symptoms.

An important methodological consideration is the temporal structure of the clinical features used in the ML analysis. Some of the variables used in the model were collected at follow‐up timepoints during the intervention period, which may capture treatment‐related effects rather than baseline risk indicators. Therefore, the present models should be interpreted primarily as classification tools for analyzing intervention outcomes rather than early screening predictors. Future research should explore baseline‐only predictive models to improve the translational value of AI‐based screening systems for menopausal psychological symptoms.

From a broader theoretical perspective, the development of AI‐assisted clinical prediction systems aligns with technology adoption theories commonly used in information systems research. For example, the TAM suggests that the adoption of AI tools in healthcare depends on clinicians’ perceptions of usefulness and ease of use. Similarly, the TOE framework highlights the role of technological capabilities, organizational readiness, and external environmental factors in shaping the adoption of digital health technologies. In this context, the proposed ML approach may serve as a supportive decision‐making tool that enhances clinical assessment of menopausal psychological symptoms. Future research may further explore the integration of AI‐based health analytics with technology adoption models to better understand the implementation of such systems in real clinical environments.

From a broader healthcare perspective, the integration of ML‐based analytical tools may contribute to improved digital health strategies for women's health. AI‐assisted systems have the potential to support clinicians in identifying psychological symptoms associated with menopause and monitoring treatment responses more efficiently. Such approaches may also enhance early screening capabilities in resource‐limited healthcare settings by providing decision‐support tools that complement clinical assessment. Policymakers and healthcare administrators may consider the potential role of AI‐driven health analytics in strengthening preventive healthcare services and supporting personalized treatment strategies for menopausal women.

Although *P. anisum* is not a psychedelic, its demonstrated effects on anxiety, depression, and stress exemplify alternative approaches that may complement traditional or psychedelic interventions. This study underscores the potential of integrating traditional herbal therapies with AI‐assisted clinical tools for mental health assessment.

### 3.6 Limitations and Future Recommendations of the Study

A key limitation of this study is the absence of an external validation cohort. Although CV provides an internal assessment of model performance, the generalizability of the models to independent populations remains untested. Future studies should validate these findings in larger and diverse cohorts to confirm the robustness and applicability of the proposed ensemble‐based ML models.

Despite encouraging findings, the study has limitations, including a small sample size, which requires justification given that it is the first clinical trial on *P. anisum* for psychological and menopausal symptoms. The short follow‐up duration limits the insights into symptom recurrence and adverse events, though extending it may lead to a higher dropout rate. Hormone profiles, such as FSH and oestradiol, were not assessed due to variations in FSH levels during the perimenopausal phase. Moreover, while AI techniques were employed for data analysis, the limitations of ML models, such as their reliance on data quality and interpretability, must be considered. The findings highlight the potential of *P. anisum* in managing menopausal symptoms, with its hormone‐balancing effects. A larger Phase III trial could further validate its safety and efficacy, especially regarding oestradiol and FSH levels. Comparing *P. anisum's* efficacy to standard treatments in menopausal women would benefit clinical practice. AI can aid in analyzing data and predicting outcomes, thereby enhancing understanding of *P. anisum's* role in menopause management. Given the small sample size (*N* = 60), the high accuracy observed in our ensemble‐based ML models should be interpreted with caution. Larger, multi‐center trials are needed to validate the generalizability of these findings to broader menopausal populations.

Another limitation of this study relates to the use of composite symptom scores derived from questionnaire instruments. Although these instruments are validated and widely used in clinical research, the aggregation of multiple items into single composite indicators may reduce sensitivity to individual symptom variations. Future studies with larger datasets may explore item‐level modeling approaches to further improve predictive accuracy.

## 4 Conclusion

The present study demonstrates that ensemble‐based machine‐learning models, particularly RF, can effectively classify intervention‐related clinical and psychological outcome patterns in menopausal women using multidimensional clinical and questionnaire‐based measurements. Combined with the clinical findings, the results provide preliminary evidence supporting the potential therapeutic benefits of *P. anisum* for improving psychological and menopausal symptoms. The machine‐learning findings should be interpreted cautiously because the feature set included post‐intervention measurements and the study involved a relatively small cohort. Accordingly, the models do not provide prospective prediction of psychological disorders or establish an early‐risk screening capability. Future research should evaluate baseline‐only predictive models in larger, independent prospective cohorts to determine whether ML can support early screening or future risk assessment of psychological symptoms among menopausal women. The three classifiers, such as RF, GB, and AB, were used. Among these, the RF classifier showed the highest performance across all metrics. It achieved an AUC of 0.877 and an accuracy of 0.917 in threefold CV. These results suggest that an RF‐based model can help in the early identification of psychological disorders in menopausal women, especially in limited clinical resources. The proposed ML framework also highlights the potential role of AI‐enabled clinical decision‐support systems in advancing digital health initiatives and improving healthcare management for menopausal women. Future research should involve larger and multi‐centered datasets to enhance the model's robustness. It is also essential to explore advanced feature selection methods and apply explainable AI methods to improve clinical interpretability. Long‐term follow‐up studies are needed to examine the lasting effects of treatment. The proposed model may be used in web‐based platforms as a supportive tool. However, it is intended to assist physicians, not to replace the professional medical prescription. While the intervention used in this study differs pharmacologically from psychedelics, the observed mental health benefits highlight the relevance of non‐conventional interventions in mental health care. Our findings provide a framework for considering herbal and alternative therapies alongside emerging psychedelic approaches.

## Author Contributions

**Md Belal Bin Heyat**: conceptualization, validation, project administration, supervision, writing – review and editing, writing – original draft. **Arshiya Sultana**: writing – original draft, validation, methodology, conceptualization. **Attiq Ur Rehman**: conceptualization, investigation, writing – original draft, writing – review and editing, formal analysis, data curation, resources. **Arfa Begum**: conceptualization, funding acquisition, validation, formal analysis, supervision. **Mohamad Sawan**: supervision, project administration, software, data curation, writing – review and editing.

## Funding

This work is supported by Westlake University (10318A992001) and the Hangzhou Postdoctoral Research Project (103120036582502).

## Conflicts of Interest

The authors declare no conflicts of interest.

## Supporting information




**Supplementary Materials**: brb371787‐sup‐0001‐SuppMat.docx

## Data Availability

All data used in this study are available upon request.
