# Supplementary material for: Ensemble‐Based Machine Learning Models for Classifying Intervention‐Related Psychological Outcome Patterns in Menopausal Women Treated With Pimpinella anisum
Source: Brain Behav. 2026 Oct 9;16(10):e71787. doi: 10.1002/brb3.71787 (PMC13653034; doi:10.1002/brb3.71787)
Supplement: Supplementary file 1 [file BRB3-16-e71787-s001.docx]

**Supplementary Material**

A data dictionary and metadata description outlining feature definitions, variable types, and preprocessing rules are provided in the supplementary material to support reproducibility of the machine learning pipeline.

**Table S1.** Feature List and Data Dictionary Used in the Machine Learning Analysis

| Feature Name | Variable Description | Data Type | Measurement / Encoding |
| --- | --- | --- | --- |
| Age | Age of participant at baseline | Numeric (Continuous) | Years |
| BMI | Body Mass Index of participant | Numeric (Continuous) | kg/m² |
| Menopausal Status | Menopause stage of participant | Categorical | One-hot encoded (Perimenopause / Postmenopause) |
| Intervention Group | Treatment assignment | Categorical | One-hot encoded (Herbal Group / Control Group) |
| DASS-21 Depression Score | Depression subscale score from DASS-21 questionnaire | Numeric (Continuous) | Total subscale score |
| DASS-21 Anxiety Score | Anxiety subscale score from DASS-21 questionnaire | Numeric (Continuous) | Total subscale score |
| DASS-21 Stress Score | Stress subscale score from DASS-21 questionnaire | Numeric (Continuous) | Total subscale score |
| MKI Total Score | Modified Kupperman Index total menopausal symptom score | Numeric (Continuous) | Total score |
| MKI Symptom Scores | Individual symptom scores from MKI questionnaire (e.g., hot flashes, insomnia, nervousness) | Numeric (Continuous) | Symptom-level scores |
| VHI Score | Vaginal Health Index score assessing vaginal health | Numeric (Continuous) | Total score |
| MS-TSQ Response | Menopause Symptoms Treatment Satisfaction Questionnaire responses | Numeric / Ordinal | Questionnaire response scale |

Note: Intervention group assignment (Pimpinella anisum intervention versus control) was used exclusively as the binary target variable and was not included among the machine-learning predictor variables. Therefore, it was not one-hot encoded as an input feature.

These features include psychometric symptom scores and demographic/clinical characteristics used as input variables for machine learning classifiers.

**Table S2.** Label Definition for Machine Learning Model

| Label Name | Description | Label Type | Encoding |
| --- | --- | --- | --- |
| Intervention Response Class | Indicates participant group assignment in the randomized clinical trial | Binary Classification | 0 = Control Group (CG), 1 = Herbal Group (HG) |

Participants were randomly assigned to either the Herbal Group (HG) receiving *Pimpinella anisum* treatment or the Control Group (CG) receiving placebo capsules.

The machine learning model predicts the group classification based on questionnaire-derived clinical and symptom variables.

**Table S3.** Preprocessing Summary (Supplementary Note)

| Step | Description |
| --- | --- |
| Missing Data Handling | Intent-to-treat analysis with Last Observation Carried Forward (LOCF) |
| Feature Scaling | Z-score normalization for continuous variables |
| Categorical Encoding | One-hot encoding for categorical variables |
| Dimensionality Reduction | Not applied due to moderate feature dimensionality |

These preprocessing steps were applied before model training to ensure standardized feature representation and improve classifier performance.
